# Assessing cardiovascular risks from a mid-thigh CT image: a tree-based machine learning approach using radiodensitometric distributions

**DOI:** 10.1038/s41598-020-59873-9

**Published:** 2020-02-18

**Authors:** Carlo Ricciardi, Kyle J. Edmunds, Marco Recenti, Sigurdur Sigurdsson, Vilmundur Gudnason, Ugo Carraro, Paolo Gargiulo

**Affiliations:** 1 Institute for Biomedical and Neural Engineering, Reykjavík University, Reykjavík, Iceland; 2 Department of Advanced Biomedical Sciences, University Hospital of Naples ‘Federico II’, Naples, Italy; 3 Icelandic Heart Association, (Hjartavernd), Kópavogur, Iceland; 4 Faculty of Medicine, University of Iceland, Reykjavík, Iceland; 5 CIR-Myo, Department of Biomedical Sciences, University of, Padova, Italy; 6 A&C M-C Foundation for Translational Myology, Padova, Italy; 7 Department of Science, Landspítali, Reykjavík, Iceland

**Keywords:** Cardiology, Biomedical engineering

## Abstract

The nonlinear trimodal regression analysis (NTRA) method based on radiodensitometric CT distributions was recently developed and assessed for the quantification of lower extremity function and nutritional parameters in aging subjects. However, the use of the NTRA method for building predictive models of cardiovascular health was not explored; in this regard, the present study reports the use of NTRA parameters for classifying elderly subjects with coronary heart disease (CHD), cardiovascular disease (CVD), and chronic heart failure (CHF) using multivariate logistic regression and three tree-based machine learning (ML) algorithms. Results from each model were assembled as a typology of four classification metrics: total classification score, classification by tissue type, tissue-based feature importance, and classification by age. The predictive utility of this method was modelled using CHF incidence data. ML models employing the random forests algorithm yielded the highest classification performance for all analyses, and overall classification scores for all three conditions were excellent: CHD (AUCROC: 0.936); CVD (AUCROC: 0.914); CHF (AUCROC: 0.994). Longitudinal assessment for modelling the prediction of CHF incidence was likewise robust (AUCROC: 0.993). The present work introduces a substantial step forward in the construction of non-invasive, standardizable tools for associating adipose, loose connective, and lean tissue changes with cardiovascular health outcomes in elderly individuals.

## Introduction

The progressive degeneration of skeletal muscle is consistently identified as an independent risk factor for significant morbidity, disability, and mortality in aging individuals^1–7^. Defined as sarcopenia, recent literature has interrogated its mediating and moderating roles in a wide range of adverse health outcomes, including its role in the etiology of cardiovascular pathophysiology. Catabolic inflammatory cytokine production and characteristic adiposity from the progression of sarcopenia have been linked with the onset of diabetes^8^, hypertension^9^, and dyslipidemia^10^ – all of which are well-established risk factors for coronary heart disease (CHD)^11^ and all-type cardiovascular disease (CVD)^12–15^. Chronic heart failure (CHF) patients frequently develop cardiac cachexia, a similar muscle wasting condition whose advanced stage has been implicated as an accelerated analogue of sarcopenic muscle degeneration^16^. Indeed, the progression of sarcopenia in older CHF patients may be considerably entangled with embedded cachexic effects^16,17^. While literature cites the associations and potential causal mechanisms between cardiovascular pathophysiology and downstream changes in skeletal muscle form and function^18^, validating standardized predictive models for these conditions remains debated. Furthermore, incorporating more nuanced quantitative methods for the non-invasive prediction of these events remains a priority in literature. Identifying such a methodology would further establish the generalizability of skeletal muscle research to the early detection of cardiovascular pathophysiology and facilitate the identification of compensatory targets for clinical intervention.

**Figure 1:**
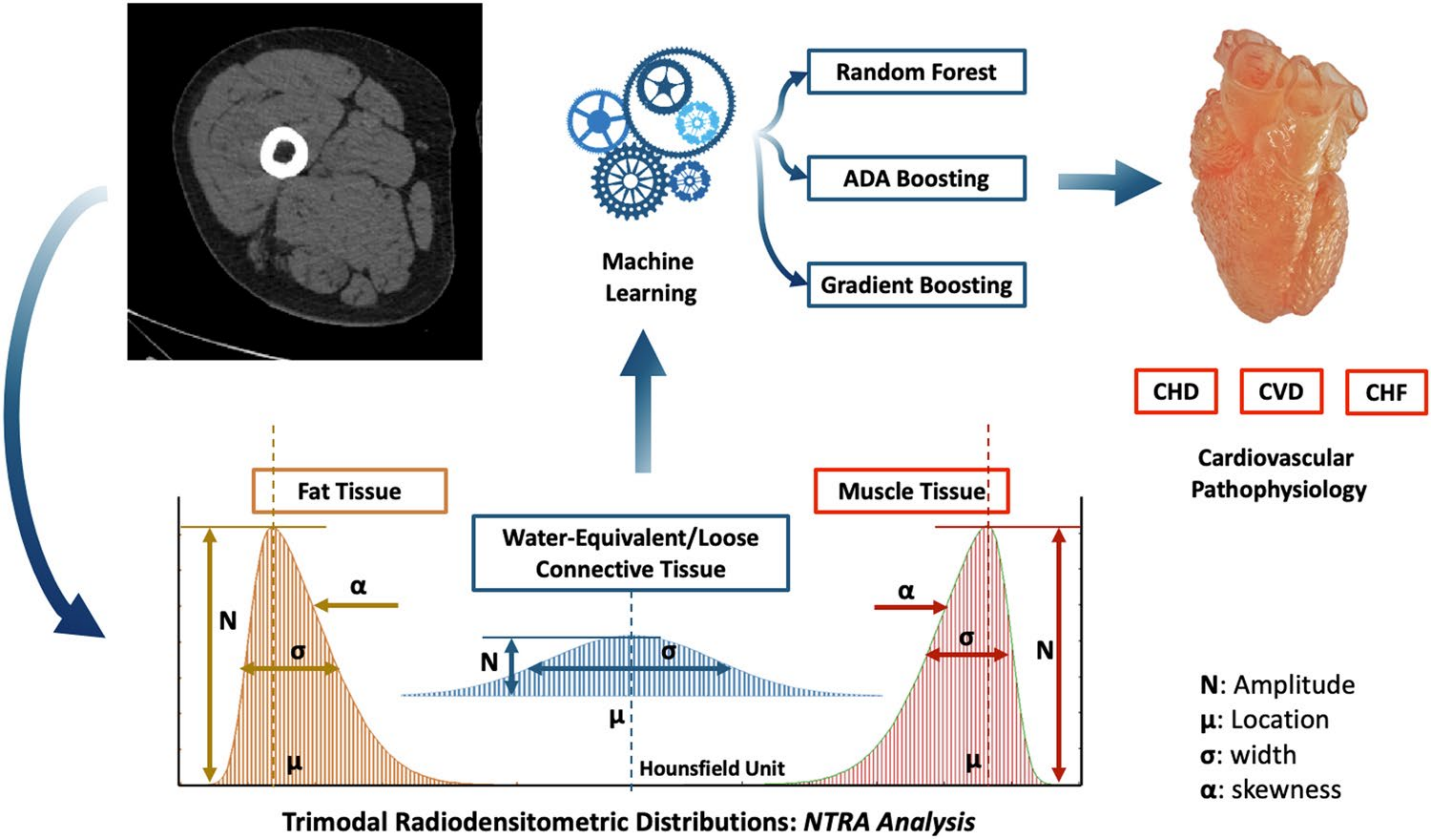
Workflow of the present study with nonlinear trimodal regression analysis parameters Gaussian distribution: from a mid-thigh CT scan, 11 radiodensitometric distributions parameter are extracted and used as features for assessing cardiovascular risks through three tree-based algorithms.

The concomitant loss of muscle mass and increase in adipose tissue in aging individuals suggest the use of quantitative imaging techniques, such as X-ray computed tomography (CT) or magnetic resonance imaging (MRI) to characterize overall changes in skeletal muscle^19–21^. Indeed, another defining characteristic of aging is the loss of muscle strength from both the reduction of dense contractile myofibers and the infiltration of non-contractile adipose tissue - a phenomenon known as myosteatosis^22^. These changes altogether present a reduction in muscle ‘quality’, which has been cited as a significant causal mechanism in the loss of muscle function - particularly when in conjunction with reduced muscle mass^13,14,23^. CT imaging has shown particular utility in quantifying these changes^20,21^. This is often performed via the use of radiodensitometric absorption values, measured in Hounsfield units (HU). Here, changes in segmented cross-sectional areas have been used to illustrate changes in volume^24–30^, and changes in average HU values have been used to illustrate changes in muscle quality^31,32^. We have recently shown the utility of modelling entire radiodensitometric distributions from CT cross-sections of the mid-thigh, highlighting the novel nonlinear trimodal regression analysis (NTRA) method^33,34^. Indeed, soft tissue HU distributions associated with cross sections from the mid-thigh can be characterized by tissue types: fat, loose connective, and lean muscle (Fig. 1). These sub-distributions are Gaussian in form and can be defined by amplitude, location, width, and skewness parameters. These parameters establish a unique 11-term soft tissue profile for each individual that can be defined using NTRA analysis for whole HU distributions^32^. In developing and using these profiles, we have demonstrated the predictive value of these parameters with functional biometrics, as well as biochemical and nutritional data from healthy aging volunteers in the longitudinal AGES-Reykjavík study. This large-scale population research study (n = 3,157) was designed to examine risk factors and disease associated with aging, including genetic susceptibility and environmental interactions.

In the present study, we compare the integration of these 11 NTRA parameters to classify elderly at risk for CHD, CVD, and CHF using multivariate logistic regression modelling and three different tree-based ML algorithms: random forests (RF), ADA-Boost (ADA-B), and gradient boosting (GB). These algorithms were applied, using regression, by Recenti *et al*.^35^ on the AGES database with the NTRA parameters to predict Body Mass Index (BMI). Figure 1 depicts this study workflow. Results from each ML model were assembled over a typology of four predictive comparisons: total classification score, classification by tissue type, tissue-based feature importance, classification by age. Further model validation was compared for each ML model using longitudinal CHF data. Results from this investigation highlight the substantial capacity of NTRA-based ML modelling to predict all three cardiovascular health outcomes; these findings are most evidenced by the high classification scores of RF models with CHF – findings which are further validated by the robust predictive performance of CHF incidence from longitudinal data. The present study altogether serves as a substantial step forward in the construction of reproducible tools for predicting cardiovascular health in elderly individuals.

## Results

### Descriptive AGES-Reykjavik statistics and NTRA parameters

Prior to the construction of logistic regression and ML models, descriptive statistics and mean NTRA parameters were assembled from the AGES-I and AGES-II databases. Table 1 contains a summary of these values. These NTRA parameters describe four fundamental features of each individual’s HU distribution: amplitude, width, location and skewness. The amplitude and width terms generally describe the summed area of each tissue type. The location parameter indicates mean tissue radiodensity, while skewness reflects the geometrical symmetry of the muscle and fat Gaussian distributions (See Fig. 1). As shown, from the total sample size of n = 3,157 subjects who were present for both studies, there were no changes in subsamples for CHD or all-type CVD (n = 628 and 753, respectively). However, the number of subjects with CHF increased from n = 59 to 183 in the five years between these datasets. Mean NTRA parameters were similar between subjects presenting with cardiovascular pathophysiology, but amplitude (*N*), location (*μ*), and width (*σ*) parameters differed somewhat for individuals with no condition.

**Table 1:** Summary statistics and nonlinear trimodal regression analysis parameters with relative standard deviation (SD) from AGES-I and AGES-II subjects by cardiac pathophysiology (coronary heart disease (CHD), cardiovascular disease (CVD), chronic heart failure (CHF), and no condition).

AGES-I Dataset						AGES-II Dataset					
*Summary measure:*		*Cardiac Pathophysiology*:				*Summary measure:*		*Cardiac Pathophysiology*:			
		*CHD*	*CVD*	*CHF*	*No Condition*			*CHD*	*CVD*	*CHF*	*No Condition*
Sample size (n)*		628	753	59	2394	Sample size (n)*		628	753	183	2322
Age: Mean (SD)		75.5 (4.7)	75.6 (4.8)	76.6 (5.3)	74.6 (4.8)	Age: Mean (SD)		80.7 (4.7)	80.8 (4.8)	82.3 (5.3)	79.7 (4.8)
Sex (Male)		419	464	34	859	Sex (Male)		419	464	96	831
Sex (Female)		209	289	25	1535	Sex (Female)		209	289	87	1491
**Mean NTRA (SD)**						**Mean NTRA (SD)**					
***Tissue:***	***Parameter:***	***CHD***	***CVD***	***CHF***	***No Condition***	***Tissue:***	***Parameter:***	***CHD***	***CVD***	***CHF***	***No Condition***
Fat	Amplitude: N	51.5 (28.5)	53.5 (29.1)	54.8 (25.8)	64.6 (33.9)	Fat	Amplitude: N	52.7 (28.9)	54.2 (29.3)	58.8 (29.6)	64.6 (33.9)
	Location: μ	−118.0 (3.8)	−117.9 (3.7)	−116.6 (4.2)	−117.8 (3.2)		Location: μ	−117.0 (5.3)	−116.8 (5.9)	−115.9 (4.5)	−117.3 (4.2)
	Width: σ	9.6 (6.8)	9.2 (6.5)	8.4 (4.8)	7.9 (5.7)		Width: σ	9.1 (6.2)	8.9 (6.0)	8.5 (5.2)	7.9 (5.6)
	Skewness: α	−2.8 (2.2)	−2.7 (2.2)	−2.3 (1.4)	−2.4 (2.0)		Skewness: α	−2.7 (2.0)	−2.6 (2.0)	−2.7 (1.8)	−2.4 (1.9)
Connective	Amplitude: N	43.6 (9.0)	43.2 (9.0)	43.2 (10.4)	41.2 (8.2)	Connective	Amplitude: N	43.9 (9.7)	43.4 (9.6)	42.2 (10.2)	41.4 (9.0)
	Location: μ	−14.5 (28.2)	−16.7 (28.3)	−21.0 (28.2)	−26.3 (28.3)		Location: μ	−17.7 (27.3)	−19.2 (27.6)	−27.7 (30.7)	−27.6 (28.0)
	Width: σ	24.3 (6.0)	24.5 (5.9)	24.9 (6.1)	25.3 (5.7)		Width: σ	23.9 (5.5)	24.0 (5.6)	22.2 (5.3)	24.9 (5.5)
Muscle	Amplitude: N	82.4 (18.9)	81.2 (18.6)	78.3 (19.7)	77.1 (17.6)	Muscle	Amplitude: N	76.3 (18.4)	75.0 (18.2)	69.8 (17.7)	72.0 (17.1)
	Location: μ	61.5 (2.7)	61.4 (2.7)	61.0 (2.9)	61.5 (2.6)		Location: μ	60.9 (2.9)	60.7 (3.0)	59.5 (3.2)	61.0 (2.8)
	Width: σ	8.5 (2.3)	8.6 (2.2)	8.9 (2.1)	8.6 (2.1)		Width: σ	9.1 (2.5)	9.1 (2.5)	10.1 (3.4)	9.1 (2.6)
	Skewness: α	2.9 (0.9)	2.9 (0.9)	3.0 (0.7)	2.8 (0.7)		Skewness: α	2.9 (0.8)	2.9 (0.8)	3.2 (0.9)	2.9 (0.8)

*Note:* *From the total sample size of n = 3,157 subjects that participated in both the AGES-I and AGES-II studies, 585 individuals presented with more than one cardiac pathophysiology.

### Logistic regression models

As described, three multivariate logistic regression models were generated for CHD, CVD, and CHF binary indicator variables using each of the 11 NTRA parameters as independent variables, and age and sex as hypothesized confounders. No discernable NTRA nonlinearity was observed from NTRA logit scatter plots, and deviance residual diagnostic plots yielded negligible heteroscedasticity and no high-leverage outliers (see Appendix A for logit plots, predicted probability curves, and deviance residual diagnostic plots). Results from each logistic regression model are assembled in Table 2.

**Table 2:** Multivariate logistic regression models for coronary heart disease (CHD), cardiovascular disease (CVD) and chronic heart failure (CHF) using soft tissue nonlinear trimodal regression analysis parameters from CT images of the mid-thigh.

*Tissue*:	*NTRA*:	*Outcome variable OR (95% CI)*:		
		CHD***	CVD***	CHF***
Fat	Amplitude: N	0.987*** (0.984–0.991)	0.989*** (0.986–0.993)	0.986*** (0.979–0.993)
	Location: μ	0.999 (0.983–1.01)	1.01 (0.992–1.02)	1.05*** (1.03–1.08)
	Width: σ	0.993 (0.966–1.02)	0.984 (0.958–1.011)	0.927* (0.868–0.981)
	Skewness: α	1.03 (0.966–1.11)	1.01 (0.950–1.08)	0.898 (0.788–1.02)
Connective	Amplitude: N	1.03*** (1.02–1.04)	1.03*** (1.02–1.04)	1.04*** (1.02–1.06)
	Location: μ	1.01* (1.00–1.01)	1.01* (1.00–1.01)	0.999 (0.988–1.01)
	Width: σ	0.970** (0.952–0.988)	0.972** (0.955–0.989)	0.935*** (0.900–0.971)
Muscle	Amplitude: N	1.00 (0.998–1.01)	1.00 (0.996–1.00)	0.982*** (0.973–0.990)
	Location: μ	0.997 (0.971–1.02)	0.986 (0.962–1.01)	0.937** (0.895–0.981)
	Width: σ	1.05 (0.997–1.11)	1.05 (0.998–1.10)	1.11* (1.02–1.19)
	Skewness: α	1.00 (0.892–1.13)	1.02 (0.915–1.14)	0.953 (0.791–1.14)

Notes: For each model, ***sex*** and ***age*** yielded strong significance (p < 0.001) as corrected confounders.

*p < 0.05; **p < 0.01; ***p < 0.001.

As evident in Table 2, the use of the 11 NTRA parameters in each logistic regression model yielded high overall model significance to each cardiac pathophysiology (p < 0.001), and both age and sex were indeed highly significant confounders (p < 0.001). However, individual-level significance from each NTRA parameter varied in specificity to each condition. Interestingly, the CHD and CVD models yielded analogously-significant NTRA parameters: fat amplitude and connective tissue amplitude, location, and width; contrastingly, nearly every NTRA parameter was significant in predicting the CHF outcome, with the exceptions of connective tissue location and both fat and muscle skewness. This indicates that the prediction of CHD and CVD using NTRA logistic regression may only require four parameters from the NTRA profile, with the connective tissue feature being a dominant independent variablesin this regard. Finally, the results associated to CHF indicates the importance of eight of the 11 NTRA parameters, with relatively shared importance from fat, muscle and connective tissue.

**Figure 2:**
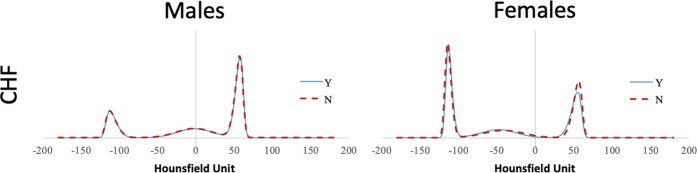
Mean Hounsfield Unit distributions for male and female subjects, with and without chronic heart failure (CHF).

As sex was a significant confounder in our logistic regression model, mean NTRA parameters were compared between male and female volunteers for each cardiac pathophysiology. Results from this comparison are shown in Table 3. From this assessment, there were few significant differences between individual NTRA parameters according to sex and condition, with the exception of muscle skewness, where female subjects presenting all three conditions had significantly higher skewness values than those without the condition. Figure 2 illustrates the mean HU distributions male and female subjects with and without CHF, as an example condition (see Appendix B for CHD and CVD distributions).

**Table 3:** Mean nonlinear trimodal regression analysis parameters from AGES-I and AGES-II subjects by sex and cardiac pathophysiology. The following convention for the p-value was employed: *p < 0.05; **p < 0.01; ***p < 0.001.

	*Tissue:*	n	Fat				Muscle				Connective		
			Amplitude: N	Location: μ	Width: σ	Skewness: α	Amplitude: N	Location: μ	Width: σ	Skewness: α	Amplitude: N	Location: μ	Width: σ
CHD	Male_Y_	838	38.3 (13.2)	−117.7 (5.3)	11.0 (7.4)	−3.1 (2.4)	86.2 (17.7)	61.4 (2.8)	8.3 (2.0)	2.8 (0.8)	45.8 (9.6)	−2.8 (20.5)	23.5 (6.1)
	Male_N_	1790	37.9 (15.1)	−117.9 (4.8)	11.5 (7.9)	−3.3 (2.9)	86.2 (18.1)	61.5 (2.8)	8.1 (2.0)	2.7 (0.7)	44.5 (9.2)	−1.7 (21.3)	23.3 (6.3)
	Female_Y_	418	79.7 (31.3)	−117.1 (2.8)	5.9 (1.2)	−2.0 (0.7)	65.5 (12.5)	60.7 (2.8)	9.8 (2.8)	3.2 (0.9)***	39.7 (7.5)	−42.6 (20.5)	25.2 (4.8)
	Female_N_	3206	79.1 (32.4)	−117.2 (3.3)	5.9 (1.9)	−1.9 (0.9)	67.7 (13.0)	61.0 (2.8)	9.4 (2.5)	3.0 (0.7)	39.4 (7.7)	−41.3 (20.5)	26.0 (5.0)
CVD	Male_Y_	928	38.4 (13.2)	−117.5 (5.8)	11.0 (7.3)	−3.1 (2.5)	85.8 (17.8)	61.4 (2.9)	8.3 (2.0)	2.8 (0.8)	45.6 (9.5)	−3.0 (20.6)	23.5 (6.2)
	Male_N_	1700	38.5 (15.2)	−118.0 (4.4)	11.5 (8.0)	−3.3 (2.9)	86.5 (18.1)	61.5 (2.7)	8.1 (2.0)	2.7 (0.7)	44.6 (9.2)	−1.6 (21.3)	23.3 (6.3)
	Female_Y_	578	78.7 (30.7)	−117.1 (3.0)	5.9 (1.3)	−2.0 (0.7)	65.7 (12.2)	60.6 (2.8)	9.8 (2.7)	3.1 (0.8)***	39.6 (7.7)	−42.0 (20.5)	25.4 (4.8)
	Female_N_	3046	79.3 (32.6)	−117.2 (3.3)	5.9 (1.9)	−1.9 (0.9)	67.7 (13.1)	61.0 (2.8)	9.4 (2.5)	3.0 (0.7)	39.4 (7.7)	−41.4 (20.5)	26.0 (5.0)
CHF	Male_Y_	130	41.3 (14.9)	−116.0 (5.4)	10.5 (6.1)	−2.9 (2.1)	79.6 (19.0)	59.7 (3.1)	8.9 (2.3)	2.9 (0.7)*	45.7 (10.4)	−7.5 (21.5)	22.9 (5.7)
	Male_N_	2509	38.3 (14.5)	−117.9 (4.9)	11.3 (7.8)	−3.2 (2.8)	86.6 (17.8)	61.6 (2.7)	8.1 (2.0)	2.7 (0.8)	44.9 (9.3)	−1.8 (21.0)	23.4 (6.2)
	Female_Y_	112	77.0 (29.1)	−116.1 (3.0)	6.1 (1.7)	−2.2 (1.1)	62.8 (13.3)	60.1 (3.1)	10.9 (3.7)	3.4 (1.0)**	38.7 (8.6)	−47.6 (23.9)	22.8 (5.6)
	Female_N_	3532	79.2 (32.3)	−117.3 (3.2)	5.9 (1.8)	−1.9 (0.8)	67.6 (13.0)	61.0 (2.7)	9.4 (2.5)	3.0 (0.8)	39.4 (7.7)	−41.3 (20.4)	26.0 (4.9)

As is evident in Fig. 2, while there are very few visual differences between HU distributions for subjects with or without CHF, but clear differences between male and female distribution curves. Here, the fat distribution is much more pronounced in female subjects, whereas the muscle distribution is more pronounced in male subjects. Likewise, the central connective tissue distribution is centered near 0 HU for males, but around −40 HU for females.

### ML models

#### Total classification scores: K-fold cross-validation and NTRA by k = 12

**Figure 3:**
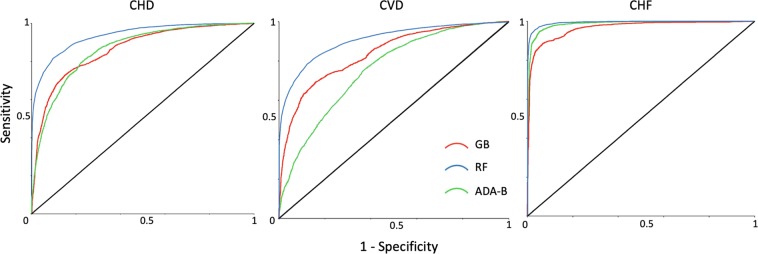
ROC curves for coronary heart disease (CHD), cardiovascular disease (CVD) and chronic heart failure (CHF) classification with K-fold cross-validation and nonlinear trimodal regression analysis by k = 12.

Prior to the generation of ML models, the smote technique was applied for all cardiac conditions to obtain a balanced dataset with an equal distribution of sick and healthy people. In this phase, the 11 NTRA parameters were employed to make the predictions with GB, RF and ADA-B. K-fold cross-validation was employed three times (k = 8,10, and 12) to compute the pathophysiology predictions; here, the 12-fold cross-validation was empirically found to be the best option for predicting all three conditions (see Appendix C for k = 8 and k = 10 results). The results from k = 12 analyses are summarized in Table 4 and the respective ROC curves are shown in Fig. 3.

**Table 4:** The 11 nonlinear trimodal regression analysis parameters were used to assess cardiovascular risks through machine learning algorithms. The evaluation metrics by cardiac pathophysiology were computed.

	Algorithm	Accuracy Mean [%]	Accuracy Max [%]	Sensitivity [%]	Specificity [%]	Recall [%]	Precision [%]	AUCROC
CHD	GB	75.9	77.7	70.0	81.7	70.0	79.3	0.864
	RF	85.0	87.4	81.7	88.4	81.7	87.6	0.936
	ADA-B	79.5	82.2	74.9	84.1	74.9	82.4	0.873
CVD	GB	73.1	75.7	67.1	79.1	67.1	76.2	0.834
	RF	82.1	83.9	78.8	85.5	78.8	84.5	0.914
	ADA-B	70.2	77.0	63.3	77.2	63.3	73.5	0.766
CHF	GB	88.6	90.3	85.0	92.1	85.0	91.5	0.962
	RF	95.9	96.5	95.0	96.9	95.0	96.8	0.994
	ADA-B	94.0	95.4	92.1	95.8	92.1	95.7	0.987

Regarding the ML analyses, CHF was classified with the highest overall scores; specifically, the RF method yielded the best results, evidenced by an accuracy of 95.9%, an exceptionally high AUCROC of 0.994, and all additional scores above 95.0%. Nevertheless, ADA-B likewise surpassed 90.0% accuracy and obtained a high AUCROC (0.987). Concerning the CHD condition, ADA-B again obtained the second highest accuracy among all pathophysiologies, and RF was again the best algorithm (85.0% in accuracy and AUCROC of 0.936). CVD was likewise accurately predicted, although the condition yielded the weakest overall results among the three, with a highest achieved predictive accuracy of 82.1% obtained from the RF method and AUCROC of 0.914.

#### NTRA-based classification by tissue type

In regarding the elaborations presented by logistic regression, ML analyses were further employed with features grouped by the three tissue types defined by their inherent NTRA parameters, as described: ***N***, ***μ***, ***σ***, and ***α*** for fat and lean muscle, and ***N***, ***μ***, and ***σ*** for loose connective tissue. Table 5 details the evaluation metrics computed per ML algorithm in this regard, defined by each tissue type and cardiac pathophysiology.

**Table 5:** The 11 nonlinear trimodal regression analysis parameters grouped by tissue type (fat, connective and muscle) were used to assess cardiovascular risks through machine learning algorithms and evaluation metrics were computed.

	Tissue	Algor.	Acc. Mean [%]	Acc. Max [%]	Sens. [%]	Spec. [%]	Recall [%]	Precision [%]	AUCROC
CHD	Fat	GB	73.8	75.2	69.6	78.0	69.6	75.9	0.828
		RF	79.6	82.2	76.1	83.1	76.1	81.8	0.884
		ADA-B	63.9	65.0	52.0	75.8	52.0	68.3	0.674
	Connective	GB	74.3	77.5	70.0	78.6	70.0	76.6	0.824
		RF	78.4	80.2	74.4	82.4	74.4	80.9	0.876
		ADA-B	63.3	65.4	56.3	70.5	56.2	65.6	0.680
	Muscle	GB	74.0	76.4	69.0	78.9	69.0	76.6	0.824
		RF	79.6	82.2	76.6	82.6	76.6	81.4	0.885
		ADA-B	63.6	66	63.3	63.9	63.3	63.7	0.673
CVD	Fat	GB	71.0	73.3	66.1	75.8	66.1	73.2	0.794
		RF	76.8	78.1	73.8	79.8	73.8	78.5	0.855
		ADA-B	61.5	64.1	50.8	72.2	50.8	64.7	0.645
	Connective	GB	71.3	74.3	66.0	76.7	66.0	73.9	0.792
		RF	76.1	78.5	71.8	80.3	71.8	78.5	0.846
		ADA-B	61.6	63.4	58.3	64.8	58.3	62.4	0.654
	Muscle	GB	70.2	72.8	65.0	75.5	65.0	72.6	0.788
		RF	76.8	78.9	73.7	79.9	73.7	78.6	0.854
		ADA-B	60.7	63.8	56.8	64.6	56.8	61.6	0.644
CHF	Fat	GB	83.0	85.0	80.2	85.9	80.2	85.0	0.918
		RF	88.4	90.0	87.3	89.4	87.3	89.2	0.956
		ADA-B	85.6	88.7	83.3	88.0	83.3	87.4	0.927
	Connective	GB	82.4	83.8	80.2	84.6	80.2	83.9	0.907
		RF	86.6	87.6	86.5	86.7	86.5	86.7	0.939
		ADA-B	82.9	85.2	80.9	84.9	80.9	84.3	0.905
	Muscle	GB	84.0	86.5	81.4	86.6	81.4	85.8	0.922
		RF	89.6	91.2	89.4	89.8	89.4	89.8	0.96
		ADA-B	87.4	89.1	85.7	89.2	85.7	88.8	0.943

When predicting cardiac pathophysiology from NTRA defined tissue type (Table 5), the best results were again obtained from RF models; CHF was predicted with mean accuracies of 88.4%, 89.6% and 86.6% for fat, muscle, and connective tissue, respectively. Fat’s features, in general, yielded the best overall predictive value for CHF. In comparison, CHD was predicted with an accuracy of 79.6% by fat and muscle, and 78.4% by connective tissue; all tissues yielded nearly identical overall predictive results. In predicting CVD, the tissues, commensurate with the previous ML results, obtained the lowest overall scores (under 80.0%). The highest model performances, in accordance with AUCROC, were achieved with the prediction of CHF, wherein all models surpassed the value of 0.9.

#### Tissue-based feature importance

**Figure 4:**
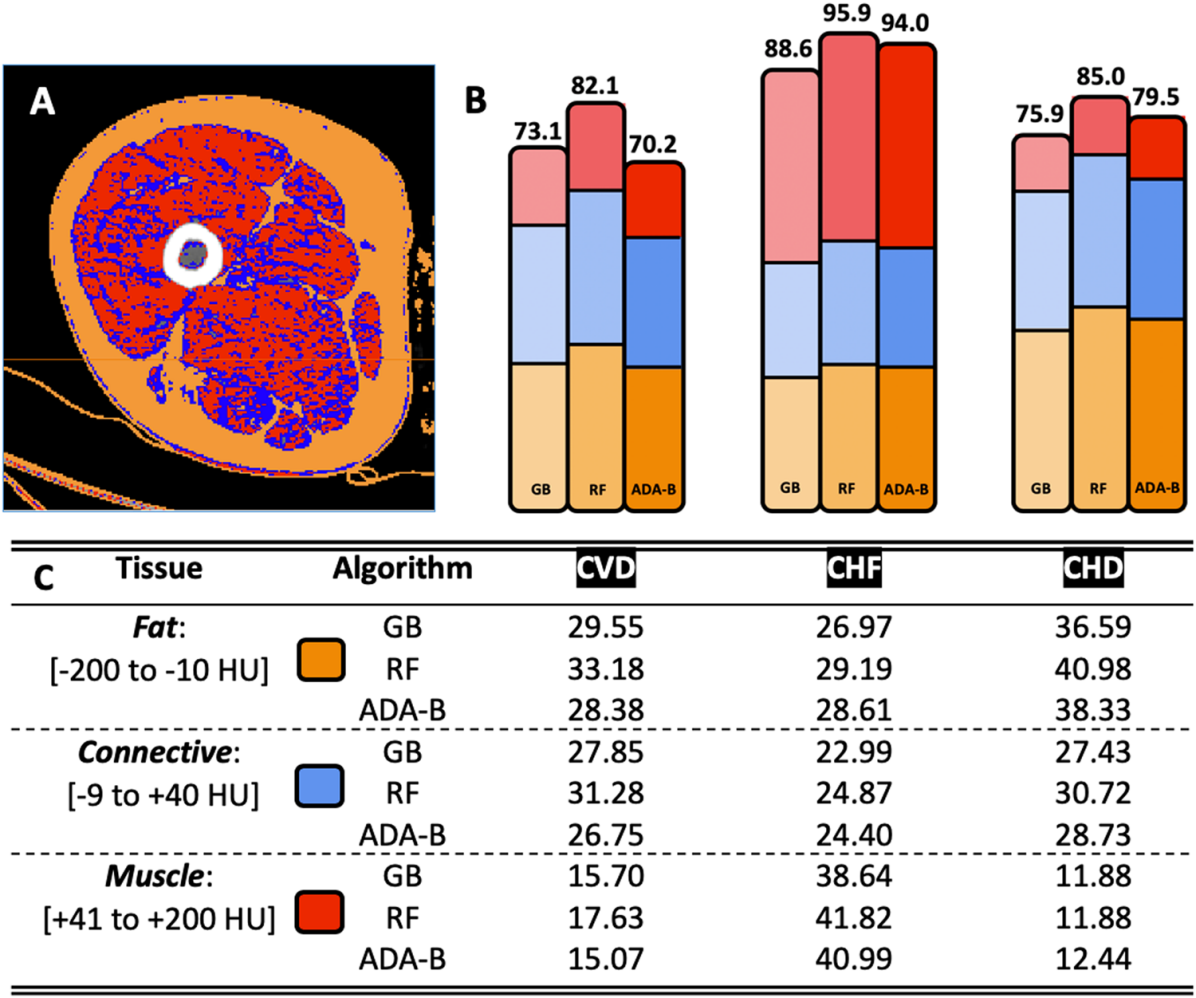
Results from tissue-based machine learning feature importance. (**A**) Example of a segmented false-color CT cross-section to illustrate the morphology of fat (orange), loose connective (blue), and lean muscle (red) tissue. (**B**) Total model accuracy (%) for each algorithm and cardiac pathophysiology, visually illustrating (with analogous colors) the compositional accuracy of each model with respect to tissue type. (**C**) Compositional accuracy (%) for each model with respect to tissue type.

Next, feature importance was computed and grouped again by tissue type defined by NTRA parameters, allowing for the comparison of the respective contributions from fat, muscle, and connective tissue NTRA values towards the accuracy of pathophysiology prediction. These tissue contributions are detailed in Fig. 4, alongside an example of a segmented false-color CT cross-section that illustrates the morphology of each NTRA tissue type.

#### NTRA-based classification by age

As logistic regression models implicated age and sex as strongly significant confounders for prediction of all three cardiac conditions, we additionally sought to illustrate whether the excellent classification scores identified in initial ML analyses held with respect to age, indicating their relative dependencies. From the original database, individuals were classified into three subgroups according to their age: 66–75, 76–84, and 85–98 years old. Results from these analyses are shown in Table 6.

**Table 6:** The 11 nonlinear trimodal regression analysis parameters were used to assess cardiovascular risks on subjects grouped by age through machine learning algorithms and evaluation metrics were computed.

	Age [years]	Algor.	Acc. Mean [%]	Acc. Max [%]	Sens. [%]	Spec. [%]	Recall [%]	Precision [%]	AUCROC
CHD	66–75	GB	79.0	81.7	73.6	84.5	73.6	82.6	0.883
		RF	87.5	90.2	83.7	91.3	83.7	90.5	0.953
		ADA-B	84.3	89.3	79.5	89.2	79.5	88.0	0.920
	76–83	GB	75.9	79.7	69.5	82.3	69.5	79.7	0.862
		RF	85.3	87.4	82.9	87.7	82.9	87.1	0.930
		ADA-B	77.0	82.3	71.0	83.0	71.0	80.7	0.836
	84–98	GB	72.8	81.1	79.4	66.2	79.4	70.2	0.825
		RF	82.0	86.2	86.3	77.7	86.3	79.4	0.908
		ADA-B	71.3	81.0	81.0	61.7	81.0	61.7	0.769
CVD	66–75	GB	76.4	79.6	70.9	81.9	70.9	79.6	0.868
		RF	85.4	88.0	81.7	89.1	81.7	88.2	0.937
		ADA-B	78.3	82.1	73.6	83.1	73.6	81.3	0.858
	76–83	GB	72.0	76.2	66.4	77.6	66.4	74.8	0.817
		RF	80.6	82.4	78.5	81.9	78.5	82.7	0.890
		ADA-B	66.1	73.2	61.1	71.2	61.1	68.0	0.725
	84–98	GB	68.9	76.2	77.2	60.6	77.2	66.2	0.786
		RF	78.0	88.9	83.7	72.4	83.7	75.2	0.875
		ADA-B	62.9	67.6	64.3	61.5	64.3	62.5	0.659
CHF	66–75	GB	93.8	95.0	91.5	96.0	91.5	95.8	0.981
		RF	97.9	99.2	97.5	98.4	97.5	98.4	0.998
		ADA-B	97.5	98.7	96.2	98.8	96.2	98.8	0.997
	76–83	GB	88.1	90.5	84.6	91.7	84.6	91.0	0.963
		RF	96.0	97.0	94.7	97.4	94.7	97.3	0.995
		ADA-B	94.2	96.4	92.2	96.1	92.2	95.9	0.986
	84–98	GB	82.6	87.8	88.2	76.9	88.2	79.2	0.921
		RF	92.6	96.4	92.8	92.4	92.8	92.4	0.981
		ADA-B	89.9	93.5	92.5	87.2	92.5	87.9	0.964

For CVD, the maximum classification accuracy and AUCROC were 82.1% and 0.914; splitting into three groups, RF kept on being the best algorithm and showed an accuracy between 78.0% and 85.4%, and an AUCROC between 0.875 and 0.937. Concerning CHD, the best accuracy and AUCROC were 85.0% and 0.937, respectively; subgrouping by age, RF obtained an accuracy above 82.0% for all subgroups and an AUCROC above 0.9 for each group. Finally, CHF showed again the best results with an accuracy range between 88.6% and 95.6% and AUCROC between 0.962 and 0.994 through RF. Despite subgrouping by age, results were still excellent, presenting an accuracy range of 92.6% to 97.9% and AUCROC between 0.981 and 0.998. These results confirm that ML classification is accurate, independent from age as a confounder, and considering the operation of these algorithms, it is further reasonable to assume an analogous classification independence from sex in prediction.

#### NTRA-based longitudinal assessment

In order to validate the ML prediction results, a cross sectional dataset obtained between AGES-I and AGES-II was used; here, only CHF was possible to assess due to no change in the number of individuals who received a CVD or CHD diagnosis between the two study timepoints.

To test the predictive potential of our ML models against the diagnosis of CHF, an incidence index was defined; here, the null condition ‘0’ was assigned as a control to subjects without CHF in either AGES-I or AGES-II, whereas ‘1’ was assigned to those without CHF in AGES-I but with the condition in AGES-II. This method thereby removed all individuals presenting CHF at both timepoints. Table 7 illustrates the results from predicting CHF incidence using each of the aforementioned ML models.

**Table 7:** The 11 nonlinear trimodal regression analysis parameters from AGES-I were used to predict the presence of chronic heart failure in AGES-II through machine learning algorithms and evaluation metrics were computed.

	Algor.	Acc. Mean [%]	Acc. Max [%]	Sens. [%]	Spec. [%]	Recall [%]	Precision [%]	AUCROC
CHF	GB	88.3	90.3	85.5	91.2	85.5	90.7	0.959
	RF	95.5	97.0	93.7	97.3	93.7	97.2	0.993
	ADA-B	94.3	95.8	96.2	92.3	92.3	96.0	0.986

As shown in Table 7, the RF method again yielded the best predictive accuracy (95.2%) and AUCROC (0.993) for the prediction of CHF incidence. In contrast, ADA-B was analogously second-best in predictive accuracy (94.3%), and GB was the least accurate of the three (88.3%). Nonetheless, each ML algorithm surpassed an AUCROC value of 0.95, as well as specificity and precision values greater than 90.0%.

## Discussion

Deleterious changes in skeletal muscle in patients with poor cardiovascular health outcomes have been discussed in literature. Patients with CHF have been shown to develop significant ultrastructural abnormalities in their skeletal muscle, suggesting poor muscle oxidative capacity as reflected by decreased exercise capacity^36,37^. Indeed, abnormal skeletal muscle function, increased thigh intermuscular fat, and reduced exercise capacity have been cited as primary chronic symptoms in heart failure patients with preserved ejection fraction (HFpEF)^38^. However, literature on the use of ML-modelling for the prediction of these conditions remains scarce, despite recent systematic review evidence that highlights its promising utility in datamining and classifying health outcomes^39,40^.

At the time of this work, only one study could be found that reports using ML-modelling of CT images to classify individuals according to cardiovascular health outcomes. In this study, coronary CT angiography images were combined with ML-modelling to develop an artificial intelligence-based imaging biomarker to predict myocardial infarction in healthy subjects^41^. However, the use of CT images of skeletal muscle for classifying cardiovascular health outcomes remains unreported. Furthermore, the methodological heterogeneity between ML-based clinical studies is generally high, as predictive parameters or ML methods remain largely study-specific and unstandardized. As such, the present work aimed to explore ML-modelling techniques to classify individuals diagnosed with CHD, CVD, and CHF using CT-based NTRA parameters as a quantitative construct for skeletal muscle health.

### Summary of main findings

From our multivariate logistic regression models, several key trends emerged when comparing the odds ratios for each significant NTRA parameter. Notably, both fat amplitude and connective tissue width were significantly and inversely-related to all three outcome conditions; this suggests that an increase in fat tissue, concomitant with a wider connective tissue distribution, may be significant protective factors against cardiovascular pathophysiology. However, an increase in fat amplitude as a protective factor is somewhat counterintuitive, as increased skeletal muscle adiposity has been readily linked with poor cardiovascular health outcomes^42^. Nevertheless, these models indicate that connective tissue amplitude is significantly and directly related to all three outcome conditions, as an accumulation of pixels at this center radiodensitometric distribution was significantly associated with the probability of CHD, CVD, and CHF. Finally, as each model was generated from the same series of NTRA parameters, it is further useful to directly compare Akaike information criteria (AIC) to resolve any differences in trade-off between model fit and complexity. AIC values for the CHD and CVD models were relatively similar (5,971 and 6,657 respectively); however, the AIC of the CHF model (1,943) indicates its comparatively high parsimony, which implicates the CHF model for having the best predictive utility amongst the three^43^.

It is critical here to discuss the salience of these NTRA parameter changes to physiological changes associated with muscle degeneration. We have previously hypothesized that the characteristic infiltration of fat into lean muscle tissue defined as myosteatosis would result in a shift of ‘pure’ fat or muscle CT pixels towards the center of the HU distribution due to radiodensitometric value averaging^34^. This could, in-turn, result in several distributional changes that may occur independently; decreases in fat and muscle amplitude, a shift in fat and muscle peak locations towards zero, an increase in connective amplitude and a decrease in its width, and increases in fat and muscle skewness magnitude. Here, we see all of these phenomena together in the logistic regression prediction of all three adverse cardiovascular outcomes, with the exception of skewness terms. Indeed, this offers a possible explanation for our aforementioned counterintuitive protective factors of increased fat amplitude and connective tissue width for all three conditions. Altogether, these results serve as strong evidence that NTRA parameters hold utility in linking subtle physiological indicators of myosteatosis with cardiovascular health. While this relationship is strong for the classification of CVD and CHD, the prediction of CHF is particularly robust.

It is likewise important to discuss the pathophysiological characteristics of the three cardiovascular outcomes utilized in this study to interrogate the particular predictive strength of CHF and relative similarity in prediction of CVD and CHD. Firstly, CVD is understood as an overarching typology of cardiovascular conditions that includes CHD alongside a host of other disease types, such as atherosclerosis or myocardial infarction^44^. As such, the comparative prediction of all-type CVD and CHD may be expected to be relatively similar. Contrastingly, CVD and CHD have been implicated as a primary etiology of CHF alongside other key comorbidities such as diabetes^45^. As such, while CHF may be a downstream consequence of CVD or CHD, its prediction likely relies on additional exogenous factors and may therefore be relatively independent. This could explain the relative similarity of significant logistic regression terms and AIC for CVD and CHD compared to CHF; furthermore, residual diagnostics and predicted probability curves (Appendix A) show striking similarities between CHD and CVD models which largely differ from CHF curves.

From our ML models, there were again similarities between the classification accuracy of CVD and CHD, while CHF classification consistently outperformed the other two conditions. Nevertheless, all three conditions yielded high overall accuracies and excellent AUCROC values, suggesting the high general utility of NTRA-based modelling for all outcomes. Regarding tissue-based feature importance (Table 4), several key insights are shown, with differences apparent between cardiovascular conditions. Firstly, fat had a predominate role in classifying CHD (41.0%), while muscle had a comparatively minor contribution (11.9%). Contrastingly, lean muscle gave the highest contribution in classifying CHF (41.0%), while connective tissue yielded the lowest contribution (24.9%). Finally, fat and connective tissue gave almost the same contribution in classifying CVD (about 33.2% and 31.3%, respectively), while lean muscle was comparatively much lower (17.6%). These condition-based differences in classification indicate the potential specificity of tissue types to each condition, further suggesting the importance of segmenting classifying parameters by these three tissue types, which is one of the key features of NTRA computational modelling.

### The value of the present work

In general, this work features several key novelties for the use of skeletal muscle to classify cardiovascular health in advanced age. Firstly, we describe the NTRA computational modelling method, wherein radiodensitometric distributions from CT image cross-sections yield 11 subject-specific soft-tissue parameters that altogether present a robust and standardizable construct for quantifying muscle degeneration. This method has shown sensitivity and specificity to lower-extremity function and nutritional parameters in previous investigations^33,34^, but the present use of these parameters to classify cardiovascular health outcomes is new. Furthermore, the present work utilizes these NTRA parameters to compare the classification accuracy of three tree-based ML model algorithms with standard multinomial logistic regression, which is again novel in the context of cardiovascular health. Finally, we validate the ML classification results using longitudinal CHF data to independently model the prediction of CHF incidence.

Altogether, a key advantage of this methodology is its derivation from CT images. As a non-invasive and standardized imaging modality that is widely utilized for diagnostic applications and pathophysiological monitoring, CT-derived HU distributions of soft-tissue radiodensity can be directly compared across clinical contexts. As such, the present use of NTRA-based classification is highly reproducible and can be readily built into existing CT analysis frameworks for patient evaluation. This tool can be further adapted into additional ML-based platforms for the detection and monitoring of adverse health outcomes in accordance with the current paradigm shift towards personalized medicine^46^. Altogether, the present work serves as a substantial step forward in the construction of reproducible tools for associating skeletal muscle changes with cardiovascular health outcomes in elderly individuals.

### Limitations

As the AGES-Reykjavik study consisted of otherwise-healthy volunteers (presenting with or without various pathologies), standard clinical measurements of key cardiac functions, such as coronary perfusion or ejection fraction measurement, were absent from the dataset. For this reason, the primary purpose of this work to test the classification of cardiac health from NTRA parameters. However, the validity of our results would be strengthened by the classification of these intermediate clinical measurements, as the outcomes of CVD, CHD, and CHF are largely heterogeneous in nature. The future use of our reported methods with clinical cardiovascular data would likewise allow for the interrogation of the causal relationship between cardiac health outcomes and changes in radiodensitometric NTRA values. Further testing of this relationship using independent patient cohorts may likewise be needed to further refine our ML models.

Although in the multinomial logistic regression there are graphical (Fig. 2) and statistical (Table 3) indications of sex differences between the NTRA distributions, particularly associated to muscle and fat amplitude, this research did not investigate deeply this theme. Thus, further studies could focus more on this direction.

Finally, while evidence for the classifying power of ML-modelling continues to grow, its literature base still lacks a standardized methodology, and the mechanisms governing some of these classifications may remain unclear. As such, exploring the contextual value of different ML-modelling algorithms remains essential.

## Materials and Methods

### The AGES-I and AGES-II database

The AGES-Reykjavík study recruited 3,316 healthy subjects from 66–98 years of age (mean: 77.46) to participate in a series of two multimetric assessments separated by approximately five years, collectively defined as the AGES-I and AGES-II database. Informed consent was obtained from all participants^47^, ethical approval for patient data acquisition was obtained by the Icelandic Science and Ethics Committee (RU Code of Ethics, cf. Paragraph 3 in Article 2 of the Higher Education Institution Act no. 63/2006), and patients’ data were acquired in accordance with relevant international regulations of both Iceland and U.S. National Institutes of Health.In addition to receiving CT scans (see ‘CT acquisition’) and having a host of nutritional, neurological, and lifestyle parameters measured or surveyed, subjects were assessed for the incidence of CVD, CHD, and CHF. Of the original recruitment, n = 3,157 subjects participated in both the AGES-I and AGES-II studies separated by five years; as new CT images and incidences of cardiovascular pathophysiology were obtained separately in both studies, the total dataset size for the present work contained 6,314 records.

### CT acquisition and segmentation

All participants in the AGES-Reykjavík database were scanned with a 4-row CT detector system at 120-kV (Sensation; Siemens Medical Systems, Erlangen, Germany) as previously described^34^. The localized scanning region extended from the iliac crest to the knee joints; prior to transaxial imaging, correct positions were determined by measuring the maximum femoral length on an anterior-posterior localizer image, followed by the localization of the center of the femoral long axis. After image acquisition, for each subject, a single 10 mm section was taken from mid-thigh, midway between the acetabulum of the hip joint and the knee joint. Pixels from this slice were then processed to obtain subject-specific distributions of radiodensitometric values across the range of −200 to 200 HU.

### Nonlinear trimodal regression analysis (NTRA)

The method utilized to computationally describe each HU distribution was a form of modified nonlinear regression analysis that has been previously described^33^. Here, each HU distribution is defined as a quasi-probability density function defined by three Gaussian distributions (two skewed and one standard):

1 $${\sum }_{i=1}^{3}\varphi (x,{N}_{i},{\mu }_{i},{\mu }_{i},{\alpha }_{i})={\sum }_{1}^{3}\frac{{N}_{i}}{{\sigma }_{i}\sqrt{2\pi }}{e}^{-\frac{{(x-{\mu }_{i})}^{2}}{2{{\sigma }_{i}}^{2}}}erfc(\frac{{\alpha }_{i}(x-{\mu }_{i})}{{\sigma }_{i}\sqrt{2}})$$

where ***N*** is the amplitude, ***μ*** is the location, ***σ*** is the width, and ***α*** is the skewness of each distribution – all of which are iteratively evaluated at each CT bin, ***x***. This trimodal definition operationalizes the hypothesis that HU distributions across segmented soft tissue represent the sum of three distinct tissue types whose linear attenuation coefficients primarily occupy specific HU domains: namely, fat [−200 to −10 HU], loose connective tissue and atrophic muscle with approximately water-equivalent absorptivity [−9 to 40 HU], and lean muscle [41 to 200 HU]. The inwardly-sloping asymmetries characterized by fat and muscle distributions can be described respectively by their positive and negative skewnesses, whereas the central ‘connective’ tissue distribution is assumed to be non-skewed. Utilizing this definition, theoretical curves can be iteratively generated for each HU distribution by employing a generalized reduced gradient algorithm via the minimization of the sum of standard errors at each CT bin value. This method thereby generates 11 NTRA parameters that are altogether unique to every individual’s CT image.

### Multinomial logistic regression models and statistical analyses

As a comparative and complimentary analysis to ML modelling, three multivariate logistic regression models were first generated using generalized linear models employing the logit link function. Classification was defined for CHD, CVD, and CHF binary indicator variables, with each of the 11 NTRA parameters taken as independent predictors, with age and sex corrected for as hypothesized confounders; in total, 62 individuals were removed due to missing pathophysiology data. Predicted probabilities curves were then generated for each model, along with scatter plots for each NTRA predictor generated against the logit for each cardiovascular outcome to identify any nonlinearity in predictor variables. Deviance residual diagnostic plots were likewise generated to assess model heteroscedasticity and identify any outliers with sufficient leverage, as defined by Cook’s distance. Next, log-odds coefficients for each NTRA parameter were exponentiated to enable the direct comparison of their contributory odds ratios for each cardiovascular pathophysiology, along with 95% confidence intervals and individual-level statistical significance. Finally, overall model significance was calculated by computing the differences in χ^2^ values between null and residual deviances; logistic regression classification accuracy for each model was later computed alongside ML models to facilitate comparison.

### ML methodologies

After computing logistic regression models to predict CHD, CVD, and CHF, three tree-based ML model algorithms were performed as a methodological comparison of prediction accuracy using the 11 NTRA parameters: random forests (RF), ADA-Boost (ADA-B), and gradient boosting (GB). First, however, the ‘Smote’ technique for achieving dataset balance and k-fold cross-validation were utilized to ascertain the optimum number of mutually-exclusive folds for ML models to train and test. Following this, the results from each ML model were assembled over a typology of five comparisons: total classification score, classification by tissue type, tissue-based feature importance, classification by age, and finally classification with longitudinal data. Each of these analyses is described in the following sections.

### Knime analytic platform

The Konstanz Information Miner (Knime) analytics platform (v. 3.7.1) was employed to conduct the ML model analyses in the present study^48^. In the Knime platform, ML analyses are managed through a comfortable and intuitive workflow by combining multiple nodes and facilitating the configuration of each parameters to optimize results. Knime was in the class of “leaders” identified by the Gartner Magic Quadrant in 2017, and its validity is widely acknowledged in literature^49^; for example, Ricciardi *et al*. employed it in a study regarding gait analysis on parkinsonian patients^50^, and Romeo *et al*. and Ricciardi *et al*. conducted a radiomics ML study using Knime^51^. Similarly to the analyses reported in our present work, Mannarino *et al*. adopted the Knime platform to perform a comparison between two SPECT imaging modalities in a cardiac study and a prediction on the follow up of patients suffering from coronary artery disease^52–54^.

### Smote

Some supervised algorithms learning (such as decision trees) require an equal class distribution to obtain better and realistic classification performance. When required for the present ML methods, ‘Smote’ (Synthetic Minority Over-sampling Technique) was employed – a technique that implements an algorithm^55^ that generates artificial data by extrapolating between a real object of a given class and one of its nearest neighbors (of the same class). It then chooses a point along the line between these two objects and determines new object attributes based upon this randomly chosen point.

### K-fold cross-validation

Finally, prior to ML modelling, the statistical procedure known as k-fold cross-validation was employed^56^; this method divides a dataset randomly into ‘k’ mutually-exclusive subsets (or ‘folds’) of equal dimension. This model is then trained and tested ‘k’ times, wherein each training is performed on different ‘k–1’ folds and tested on fold ‘k’. The cross-validation estimate of accuracy is defined as the overall number of correct classifications divided by the number of instances in the dataset.

### Machine learning tree-based algorithms

The ensemble learning techniques of randomization, bagging and boosting were applied on decision tree. On the one hand, decision tree is the easiest algorithm known in literature and it does not need the normalization of the six thousand patients in AGES dataset; on the other hand, it is a weak and instable learner and ensemble techniques are useful to improve the performance of weak and instable algorithms and reduce the noise in AGES dataset.

The first ML method employed for this work was the Random Forests (RF) ensemble learning method, which features Decision Trees that share identical basic properties and the capacity to avoid overfitting^57,58^. Each tree is learned on its own, but some randomization is injected into this phase to reduce the variance of the predictions; this is performed by subsampling the AGES on each iteration to get a different training set or consider different random subsets of the 11 NTRA to split upon at each tree node. To make a prediction on a new patient, RF aggregates predictions from all their decision trees by a majority vote.

The second ML method we utilized was Ada-Boost (ADA-B) – another ensemble method belonging to the boosting family, whose core principle is the strengthening of weak learners^59^. ADA-B training selects only the NTRA parameters that improve the predictive power of the model, reducing model complexity in terms of dimension and thereby improving execution time. Data modifications at each boosting iteration consist of applying weights to every training sample, setting them such that the first step consists of training the learner on the original training data. For all other successive iterations, sample weights are modified, and the learning algorithm is applied again to the data with its new weight. At a given step, patients used for training that were wrongly predicted by the boosted model at the previous step have their weights increased, whereas these weights are decreased for examples that were predicted correctly. As iterations proceed, patients that are difficult to predict/diagnose receive ever-increasing influence. Each sequential weak learner is then forced to concentrate on patients that are previously missed.

The third ML method utilized for the present work was Gradient Boosting (GB); this method produces competitive, highly robust, interpretable procedures for both classification and regression, which is especially appropriate for mining sub-optimally clean data. Our implementation follows the algorithm of Friedman^60^. Not only does this method exploit randomization and bagging principles, but it also includes a special form of boosting to build an ensemble of weak models (in this case, decision trees).

### Evaluation metrics

A wide range of evaluation metrics are well known in literature^61^, but the following six were employed for this study:

- *Accuracy*: the number of correct predictions over their total number.
- *Recall*: the fraction of positive patterns that are correctly classified.
- *Precision*: the positive patterns correctly predicted over the total number of predictions in a positive class.
- *Sensitivity*: the number of true positives over the sum of true positives and false negatives.
- *Specificity*: the number of true negatives over the sum of true negatives and false positives.
- *AUCROC*: Area Under the Curve Receiver Operating Characteristic – probabilistic performance measurement of classification.

## Supplementary information

Supplementaryinformation

## Data Availability

The AGES I-II dataset cannot be made publicly available, since the informed consent signed by the participants prohibits data sharing on an individual level, as outlined by the study approval by the Icelandic National Bioethics Committee. Requests for these data may be sent to the AGES-Reykjavik Study Executive Committee, contact: Ms. Gudny Eiriksdottir, gudny@hjarta.is.
