# Supplementary material for: Assessing cardiovascular risks from a mid-thigh CT image: a tree-based machine learning approach using radiodensitometric distributions
Source: Sci Rep. 2020 Feb 18;10:2863. doi: 10.1038/s41598-020-59873-9 (PMC7029006; doi:10.1038/s41598-020-59873-9)
Supplement: Supplementary file 1 — Supplementaryinformation [file 41598_2020_59873_MOESM1_ESM.docx]

| **Assessing cardiovascular risks from a mid-thigh CT image: a tree-based machine learning approach using radiodensitometric distributions** |
| --- |
|  |
| **Carlo Ricciardi^1,2^, Kyle J. Edmunds^1,3^, Marco Recenti^1^, Sigurdur Sigurdsson^4^, Vilmundur Gudnason^4,5^, Ugo Carraro^6^, and Paolo Gargiulo^*1,7^**  ^1^ Institute for Biomedical and Neural Engineering, Reykjavík University, Reykjavík, Iceland  ^2^ Department of Advanced Biomedical Sciences, University Hospital of Naples ‘Federico II’, Naples,Italy  ^3^ Department of Social Policy and Intervention, University of Oxford, Oxford, United Kingdom  ^4^ Icelandic Heart Association (Hjartavernd), Kópavogur, Iceland  ^5^ Faculty of Medicine, University of Iceland, Reykjavík, Iceland  ^6^ IRCCS Fondazione Ospedale San Camillo, Venice, Italy  ^7^ Department of Rehabilitation, Landspítali, Reykjavík, Iceland |

# Appendix A

The following figure illustrates logit plots and predicted probabilities curves for the logistic regression results in the present work.


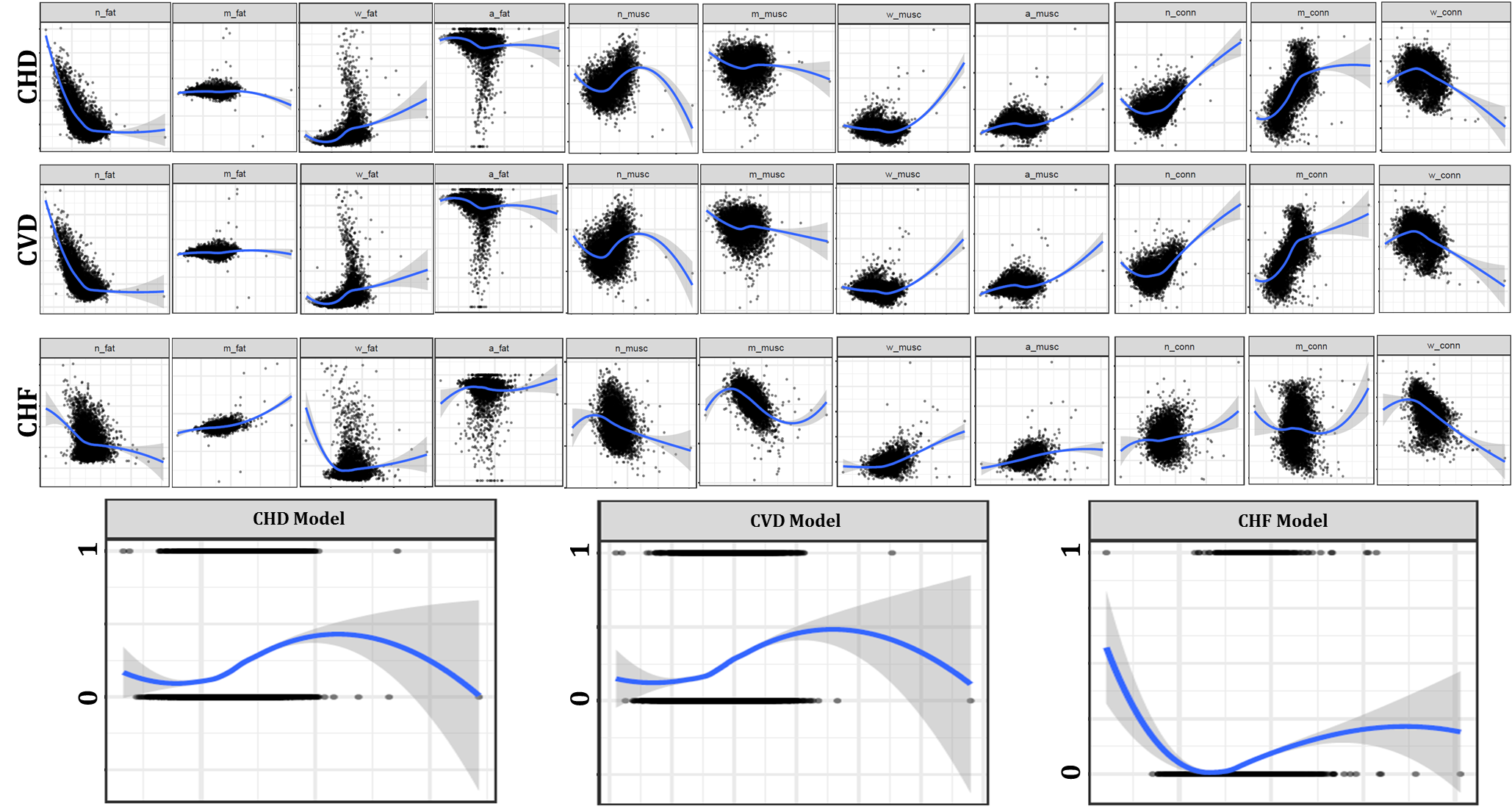


**Figure A1**. Logit plots (top rows) and predicted probabilities curves (lower row) for the logistic regression results in the present work.

# Appendix B


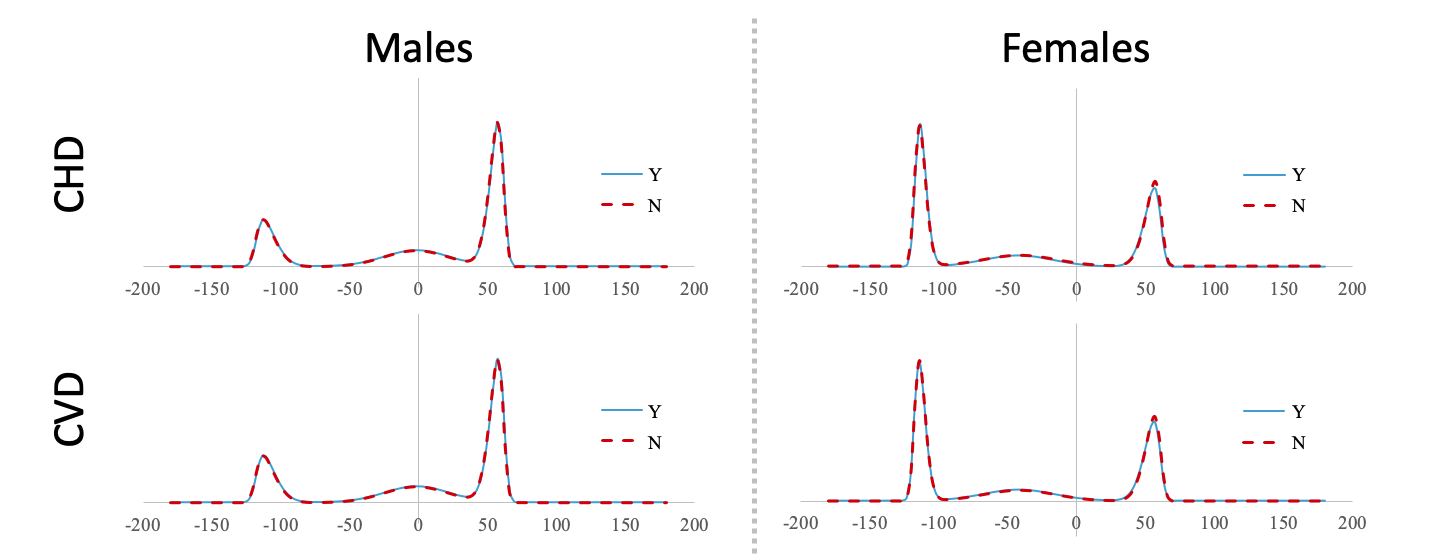


**Figure B1**. Mean HU distributions for male and female subjects with and without CHD and CVD.

# Appendix C

Results of the pathophysiology predictions with cross-validation with k=8,10.

| **Appendix C.** K-fold=8 | | | | | | | | |
| --- | --- | --- | --- | --- | --- | --- | --- | --- |
|  | **Algorithm** | **Accuracy Mean [%]** | **Accuracy Max [%]** | **Sensitivity**  **[%]** | **Specificity**  **[%]** | **Recall**  **[%]** | **Precision**  **[%]** | **AUCROC** |
| **CHD** | GB | 76,2 | 77,4 | 70,6 | 81,8 | 70,6 | 79,5 | 0,865 |
|  | RF | 84,6 | 86,2 | 81,3 | 88 | 81,3 | 87,1 | 0,932 |
|  | ADA-B | 76,6 | 80,4 | 70,4 | 82,8 | 70,4 | 80,4 | 0,838 |
| **CVD** | GB | 72,5 | 74,3 | 66,9 | 78 | 66,9 | 75,3 | 0,832 |
|  | RF | 81,8 | 82,9 | 78 | 85,6 | 78 | 84,4 | 0,91 |
|  | ADA-B | 68,5 | 74,1 | 60,7 | 76,2 | 60,7 | 71,9 | 0,754 |
| **CHF** | GB | 88,3 | 89,7 | 85,1 | 91,4 | 85,1 | 90,8 | 0,961 |
|  | RF | 95,6 | 96,8 | 94,4 | 96,7 | 94,4 | 96,7 | 0,993 |
|  | ADA-B | 93,8 | 94,7 | 91,8 | 95,8 | 91,8 | 95,6 | 0,961 |

| **Appendix C.** K-fold=10 | | | | | | | | |
| --- | --- | --- | --- | --- | --- | --- | --- | --- |
|  | **Algorithm** | **Accuracy Mean [%]** | **Accuracy Max [%]** | **Sensitivity**  **[%]** | **Specificity**  **[%]** | **Recall**  **[%]** | **Precision**  **[%]** | **AUCROC** |
| **CHD** | GB | 76,8 | 78,6 | 71 | 82,7 | 71 | 80,4 | 0,873 |
|  | RF | 85 | 86,8 | 81,4 | 88,6 | 81,4 | 87,7 | 0,936 |
|  | ADA-B | 78,5 | 80,8 | 73,6 | 83,5 | 73,6 | 81,7 | 0,858 |
| **CVD** | GB | 72,7 | 75,3 | 67,3 | 78,1 | 67,3 | 75,5 | 0,824 |
|  | RF | 82 | 83,7 | 77,9 | 86 | 77,9 | 84,8 | 0,913 |
|  | ADA-B | 69,6 | 72,7 | 62,5 | 76,7 | 62,5 | 72,9 | 0,757 |
| **CHF** | GB | 88,3 | 89,3 | 85,3 | 91,3 | 85,3 | 90,7 | 0,96 |
|  | RF | 95,9 | 96,4 | 94,8 | 97 | 94,8 | 97 | 0,994 |
|  | ADA-B | 93,9 | 94,7 | 91,9 | 96 | 91,9 | 95,8 | 0,987 |
